# Genetic changes in larval oysters are more abundant and dynamic than can be explained by rare events or error: a response to Hedgecock (2022)

**DOI:** 10.1098/rspb.2022.0197

**Published:** 2022-06-08

**Authors:** Evan Durland, Pierre De Wit, Chris Langdon

**Affiliations:** ^1^ Nofima Ås, AS, Norway; ^2^ Marine Sciences, Goteborgs Universitet, Strömstad, Sweden; ^3^ Department of Fisheries, Oregon State University College of Agricultural Sciences, Newport, OR, USA

**Keywords:** oysters, poolseq, genetic load, balancing selection, larval development

## 1. Introduction

In our recent paper [1], we reported surprisingly dynamic patterns of allele frequency change over larval development of Pacific oysters (*Crassostrea gigas*). In brief, we found that 27% (*n* = 127) of all loci with significant temporal changes had bi-directional trajectories of minor allele frequencies (MAF). The majority (62%; *n* = 282) of all loci with any changes in MAF during larval development had subsequent changes in the opposite direction, balancing the overall distortion in MAF across this period. We used these and other findings to propose a hypothesis of temporally balancing selection in developing oyster larvae whereby the variable morphological landscape of larval development has potentially heterogeneous selective effects on genes associated with many loci in the oyster genome.

In his comment, Hedgecock [2] objects to our interpretation of the results of our study, postulating that the dynamic patterns of MAF changes we report are confounded by complex genomic architectures, variability in parental reproductive success, and methodological issues. Some of the points he raises are conceptually valid, but they ignore the design of our study and the nature of our findings. Moreover, Hedgecock presents our findings and interpretations as disagreeing with those of previous studies investigating genetic changes in larval oysters, while we contend that no such fundamental conflict exists. Below, we respond to these critical issues raised by Hedgecock.

## 2. Genomic architecture

Hedgecock asserts that contrasting genomic architectures among parents (i.e. alternate linkage between the marker and causal gene variants) is one way that we have misinterpreted bi-directional changes in MAF. The two examples he provides to illustrate his point (table 1*a*,*b* in [2]) are, in our view, insufficient to explain the magnitude of changes in allele frequencies we document. The first example (table 1*a* in [2]) proposes a scenario with alternate linkage architectures which mask, not exacerbate, changes in allele frequencies. The second example (table 1*b* in [2]) is more relevant but, notably, demonstrates a very minor (approx. 5%) change in MAF, which is significantly less than the approximately 20–40% change we observed for many loci.

More broadly, these examples have limited relevance to our study when population structures are taken into account. Both scenarios in Hedgecock's example [2] are premised on a ‘pool' of only two full-sibling families with linkage associations that are neatly paired and contrasted. Our population, on the other hand, was composed of 95 families produced from a factorial cross (5 males × 19 females). In this context, the overall potential influence of these alternate linkage associations is very low due to the rarity of individual detrimental mutations [4] and the combined improbability of alternate linkage associations for these rare variants across multiple parents. We simulated this extensively and found that even in the most liberal scenarios the overall effect on MAF trajectories is negligible (see https://github.com/E-Durland/balancing-oysters). Nonetheless, we acknowledge in our discussion that multiple genes per marker may account for some of the changes we observed. We contend, however, that the magnitude and abundance of bi-directional changes in our study cannot reasonably be explained by these scenarios alone.

## 3. Variability in parental reproductive success

In a composite pool of larval oysters, the relative composition of families is expected to change across developmental time in response to genetic as well as non-genetic factors. Hedgecock [2] proposes that previous estimates of unequal reproductive success in oysters [5,6] violate neutral assumptions of even population structure and represents a fatal flaw in our interpretations. We make no such claim or assumption and, in fact, our interpretations depend upon unequal survival of oyster families as the mechanism driving changes in MAF. Further, the evidence cited by Hedgecock [2] largely refers to estimates of reductions in effective population sizes between generations, not within the larval period itself. An exception to this cited work is that of Boudry *et al*. [7] who reported an approximately 20% (not ‘30% or more') reduced effective population size when gametes from all parents were simultaneously mixed together to produce offspring; however, none of the parental contributions was lost when paired crosses were carried out separately before pooling zygotes, as was performed in our study. Second, in a broad factorial cross, maternal effects such as egg quality will be distributed among many families and genotypes thereby generalizing their effects [8] but not removing them entirely. Maternal effects are expected to impact larval survival disproportionately early in larval development (less than 6 days post fertilization) when larvae are partially dependent on egg nutrient reserves [9]; however, we observed most genetic changes in late larval development (10–22 days), in agreement with findings of Plough & Hedgecock [10].

Lastly, only changes in allele frequencies that were shared among five replicated cultures were included in this analysis, reducing the chances that random losses of families among cultures would have had a significant effect on observed patterns. Variation in allele frequency estimates among culture units (encompassing biological and technical variation) was surprisingly modest (see https://github.com/E-Durland/poolseq_variance for more details). Furthermore, our simulation algorithm (https://github.com/E-Durland/Genotype_forecaster) inherently accounted for unexpected variance in empirical estimates with a built-in error tolerance (10% as default) and repeated simulations to generate a range of possible scenarios. While variability in reproductive success of parents cannot be directly diagnosed with pooled DNA sequencing of the larvae, evidence of this possible complication is sparse but is accounted for with our analytical approach and, ultimately, is not foundational to our analyses.

## 4. Methodological issues

One of the central assumptions for pooled DNA analyses is equal template (DNA) contribution of individuals to the common pool. Template bias may arise due to numerous biological and technical reasons; however, the patterns of changes in allele frequencies that we document cannot be explained by template bias alone. First, a 4 : 1 ratio of maternal to paternal DNA in fertilized egg samples, due to retention of polar bodies, would not affect observed genetic changes past day 2. Furthermore, this factor can also be removed as a possible source of error because polar bodies I and II are released at approximately 15 and approximately 50 min post-fertilization [11] while we sampled embryos for sequencing at approximately 5 h post-fertilization.

Second, variation in larval size was small to moderate (table 1) with the greatest variance observed late in larval development (days 16 and 22).

**Table 1.** Larval size across the developmental period. Recreated from Durland *et al.* [3].

days post fertilization	larval size (µm ± s.d.)
2	78.4 ± 4.8
6	111.4 ± 7.4
10	114.8 ± 16.7
16	222.1 ± 53.0
22	439.6 ± 112.6

Template bias is expected to skew MAF of a pooled sample towards larger individuals, the effect of which should be positively correlated with variation in size. Under this scenario, we would expect to see consistent (uni-directional) changes in allele frequency favouring larger genotypes from day 10 to 22 in our study. By contrast, we see the most abundant *bi-directional* changes during this period of larval development (fig. 2 in Durland *et al*. [1]). For template bias to account for these changes, it would be necessary that groups of the largest larvae at three subsequent time points (day 10, 16 and 22) would each be genetically dissimilar from the previous one, driving reversals in MAF changes, on a population level, between each time point. Mortality during this time would also have to be genetically negligible relative to the effects of template bias (which is contrary to previous findings, e.g. those of Plough *et al*. [12]) and all changes would have to have been consistent across five biological replicates. This an extremely unlikely scenario but one that would still be consistent with our broader hypothesis that genetic determinants of fitness are temporally heterogeneous and do not favour a single allele at all loci during larval development.

## 5. Not in conflict with previous studies

Our study was not designed to identify deleterious alleles in a cohort of oyster larvae but to evaluate how genetic markers across the genome change during larval development. This type of study is lacking in the long history of genetic investigations of larval oysters, which is understandable given the difficulties in genotyping larvae. Recent studies with urchins [13] and mussels [14] have adopted a pooled sequencing approach for detecting genetic changes larval populations but did not evaluate temporal patterns in great detail. It is not too surprising that our study—with new methods, enhanced replication, and increased genomic and temporal resolution—generated findings with no close comparison in the published literature. Contrary to Hedgecock's assertions, however, our findings do not contradict previous estimates of genetic load (e.g. Plough *et al*. [4]), variance in reproductive success (e.g. Boudry *et al*. [7]), or estimates for low effective population sizes in oysters (e.g. Hedgecock *et al*. [15]). In fact, in an investigation of the timing of ‘expression' of genetic load during larval development, Plough & Hedgecock [10] also had a high proportion of markers which displayed bi-directional changes in MAF. The limited number of markers in that study suitable for time-series analysis limited the reporting of these observations to ‘the two homozygous genotypes became deficient at different time points' but the patterns are evident in their data nonetheless (see our re-analysis of their data here: https://github.com/E-Durland/MAF_v_genotypes). It is also worth noting that, unlike in our study, none of the more recent investigations of genetic load in oysters (e.g. [4,10,16]) analysed these changes in replicated larval cultures, making it impossible to disentangle genetic from environmental effects in their analyses. This complication is worthy of consideration given the variability in survival and sensitivity of larval oysters to environmental stressors [3,17] and the often vague reporting of larval rearing conditions in these previous studies.

Our findings do not contradict genetic load as an important factor in causing larval oyster mortality nor challenge theories of purifying selection. What we do demonstrate is that the temporal ‘expression' of genetic load across larval development is not as straightforward as previously expected. In our discussion, we propose several mechanisms which can account for the observed dynamic patterns of genetic change and discard none of these alternatives outright. The persistence of negative mutations through partial dominance and survival of heterozygotes, which Hedgecock highlights, is entirely consistent with our findings, but described more fully in our study than has been previously possible. We contend that rather than some instantaneous or fixed genetic effect, it is temporally offset patterns of selection against homozygotes which generate an overabundance of heterozygotes for many loci at the conclusion of larval development. With a composite population and pooled DNA samples, we cannot unequivocally diagnose causative genetic mechanisms but the overall scope of our findings cannot, in our view, be easily explained by the conflating factors suggested by Hedgecock [2]. Although our hypothesis of temporally balanced selection is novel, it remains to us, the most parsimonious explanation of our findings and adds to, rather than challenges, much of the work which Hedgecock suggests we disregard. Initially, new ideas often appear incompatible with existing paradigms, but they should not be dismissed as ‘neither warranted or needed' without providing strong evidence that they are invalid. As with all new hypotheses, we hope and expect future research, fortified with individual genotypes and increased genomic coverage, will either support or refute our interpretation of the results of this study—in either case, advancing our knowledge of selective pressures affecting development of the early life stages of oysters, and other organisms.

## Data Availability

This article has no additional data.
